# The observation during small incision lenticule extraction for myopia with corneal opacity

**DOI:** 10.1186/s12886-017-0474-7

**Published:** 2017-05-25

**Authors:** Shaowei Zhang, Haipeng Xu, Ke Zheng, Jing Zhao, Weijun Jian, Meiyan Li, Xingtao Zhou

**Affiliations:** 1 Eye and ENT Hospital of Fudan University, Myopia Key Laboratory of the Health Ministry, Shanghai, China; 2 Department of ophthalmology, Xiangyang Central Hospital, Affiliated Hospital of Hubei University of Arts and Science, Xiangyang, China; 3 Department of Ophthalmology, Eye and ENT Hospital of Fudan University, Myopia Key Laboratory of the Health Ministry, No. 83 FenYang Road, Shanghai, 200031 China

**Keywords:** Small-incision lenticule extraction (SMILE), Corneal opacity

## Abstract

**Background:**

To evaluate the feasibility and efficacy of small incision lenticule extraction (SMILE) in the treatment of myopia with corneal opacity.

**Methods:**

To evaluate the treatment of myopia with corneal opacity, 9 patients (4 males, 5 females) who underwent SMILE were enrolled in this prospective clinical study. One eye of each patient was treated. The results of laser scanning and lenticule extraction were observed during the surgery, and the surgical videos were again reviewed after surgery. Uncorrected distance visual acuity (UDVA), corrected distance visual acuity (CDVA), and spherical equivalence (SE) were noted at 1 month after surgery. The depth and density of the corneal opacities were measured by anterior segment optical coherence tomography (AS-OCT) and the Pentacam anterior eye segment analyzer.

**Results:**

All procedures were uneventful and no intraoperative complications were observed. At 1 month after surgery, the UDVA of all patients was 20/25 or better and no patients lost Snellen lines. The mean safety and efficacy indexes were 1.10 ± 0.24 and 1.08 ± 0.16, respectively, at 1 month postoperatively. The mean postoperative spherical equivalent was 0.27 ± 0.23 diopter (D). All eyes were within ±0.75 D and 8 eyes (88.9%) were within ±0.50 D. There was no eccentric corneal topography or abnormal morphology in the corneal caps. The corneal opacities of all patients were within the optical zone. The mean preoperative depths in the deepest areas of corneal opacity were 152 ± 38 μm (range: 86–217 μm); at 1 month after surgery (*P* < .01), they were 117 ± 28 μm (range: 86–189 μm). The preoperative maximum density of corneal opacity was 48.5 ± 20.7 (range: 20.4 to 85.8); at 1 month after surgery (*P* > .05), it was 49.8 ± 26.7 (range: 19.8 to 82.5) at 1 month after surgery.

**Conclusion:**

Patients with corneal opacity can be successfully treated with the SMILE operation. The short-term outcome was good, however the long-term results need further study.

**Trial registration:**

The trial registration number: ChiCTR-ONRC-13003114, Date of Last Refreshed on: 2016-01-27, Date of registration on 2013-03-17(retrospectively registered).

## Background

The femtosecond (FS) laser is a near-infrared pulsed laser that can photodisrupt the transparent cornea with great surgical precision [1]. It was introduced to corneal refractive surgery in 1999 and has since gained widespread acceptance [2, 3]. Recently several studies have shown excellent safety, efficacy, predictability, and visual quality after small incision lenticule extraction (SMILE), which is performed using the VisuMax FS system [4–9]. While the FS scanning quality of lenticule is important for the postoperative visual outcomes [10], presence of corneal opacity will result in decreasing FS energy and poor scanning [11]. Therefore it is of great clinical value to study the effect of lenticule scanning in patients with corneal opacities. In this study, we evaluated the feasibility of the SMILE procedure in patients with corneal opacities and its efficacy at 1 month of follow-up.

## Methods

### Subjects

This prospective study was performed at the Department of Ophthalmology, Eye and ENT Hospital of Fudan University, Shanghai, People’s Republic of China. Myopic patients with corneal opacities within the optical zone were recruited; those with corneal opacities underneath the intended lenticule were excluded. The study was designed in conformity with the Declaration of Helsinki and was approved by the ethics committee of the Eye and ENT Hospital of Fudan University. All patients gave informed consent and received the SMILE procedure.

### Preoperative assessment

Each patient had a full eye examination as part of a routine preoperative assessment, which included uncorrected distance visual acuity (UDVA), corrected distance visual acuity (CDVA), manifest and cycloplegic refractions, length of axis, diameter of scotopic pupil, white-to-white corneal diameter (W-W), intraocular pressure (IOP), corneal thickness, corneal tomography, slit-lamp microscopy, and dilated indirect fundoscopy. The depth of the corneal opacity was measured using AS-OCT. The densities and positions of the corneal opacities were measured manually in each patient using the Scheimpflug tomography system. The maximum and minimum densities of each central corneal opacity were recorded in terms of gray values from 0 to 100, representing the range from a clear (value 0) to completely opaque (value 100).

### Surgical technique

The VisuMax FS laser system (Carl Zeiss Meditec AG, Berlin, Germany) was used to perform the SMILE procedure, each performed by the same surgeon (XZ). The following laser parameters were used: 500-kHz repetition rate, 180-nJ pulse energy, 120-μm cap thickness, 7.5-mm cap diameter, and 6.4- to 6.7-mm lenticule diameter according to the diameter of the scotopic pupil and manifest refraction. A side cut of 2 mm at the superior 12-o’clock position was created in all patients, with spot and track spacing of 4.5 μm for the cap bed, 2.5 for the cap sidecut, and 2.0 μm for the lenticule sidecut.

### Surgical observation

During the creation of the lenticule, the results of laser scanning were evaluated to determine whether intraoperative complications such as dark spots, opaque bubble layers (OBLs), etc. had been observed. During dissection and removal of the lenticule, the results of the procedure were evaluated to determine whether intraoperative complications such as lenticule rupture or tearing were observed. The surgical video was evaluated again after surgery.

### Data analysis

All patients were examined at 1 month of follow-up. The data were analyzed using SPSS 20.0 statistical software (IBM Institute, Inc., Cary, NC), and the statistical significance level was set at a *P* < .05.

## Results

**Fig. 1:**
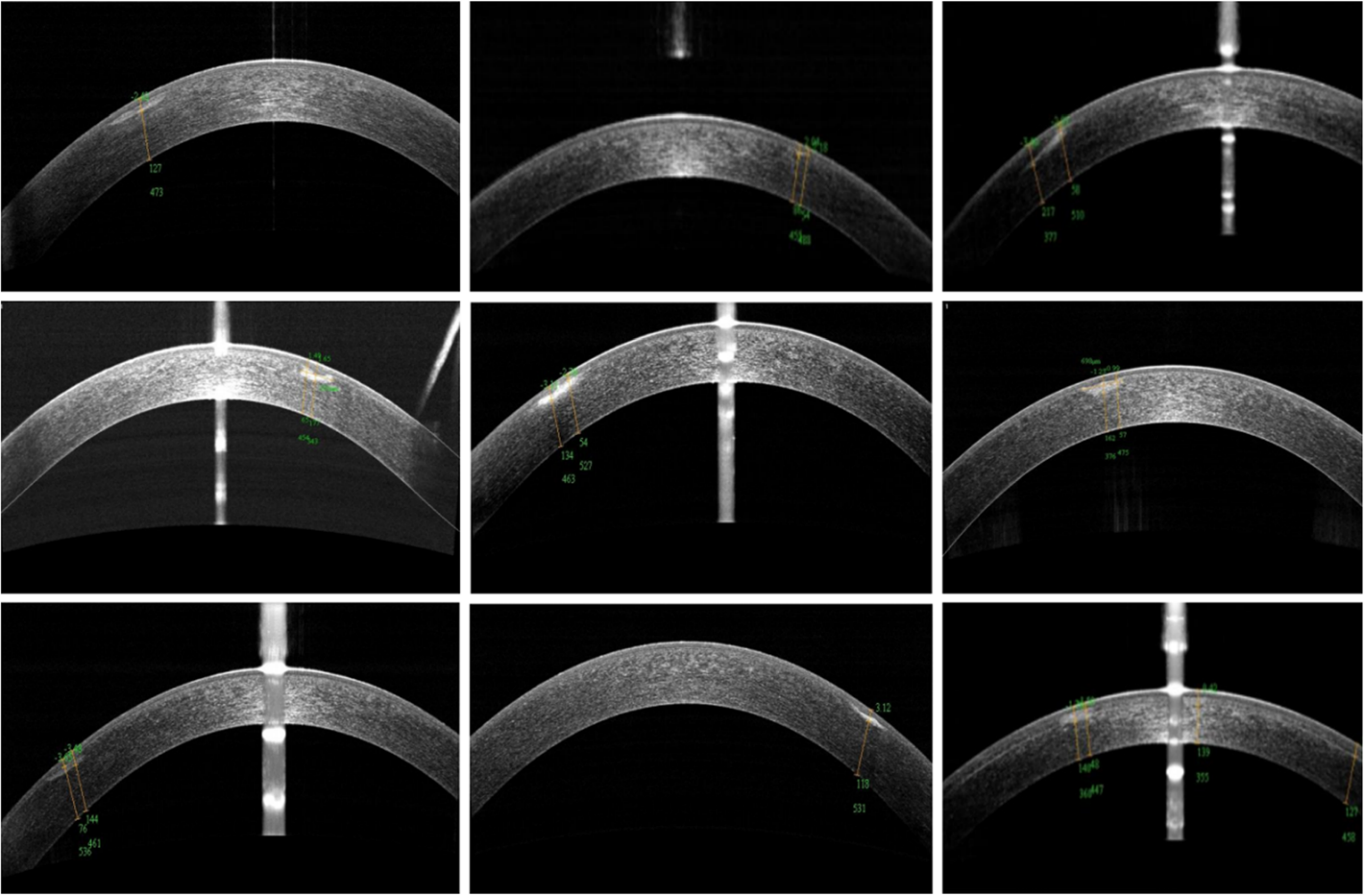
The depths of corneal opacities in each patient derived from AS-OCT examination

The mean age and preoperative spherical equivalent of patients were 34.11 ± 5.18 years (range: 19 to 42 years) and −5.56 ± 2.97 diopters (D), respectively. The corrected distance visual acuity (CDVA) of all patients was 20/20 or better. The corneal opacity of all eyes was within the optical zone. Table 1 shows the demographic data of the study population. Figure 1 shows the depth of corneal opacity from AS-OCT before surgery for each patient. There were two areas of corneal opacity within the optical zone of one patient, and there were 4 patients with a history of keratitis, 2 patients with a history of corneal foreign bodies, and 3 patients without any definite history of ocular pathology or surgery.

**Table 1:** Study demographics

Patient No.	Gender	Age	Preoperative refraction and CDVA	Depth of corneal opacity (μm)	Area of corneal opacity (mm^2^)	Density of corneal opacity
1	Male	33	−2.25/−0.25x70 = 1.2	78 ~ 127	1.223	27.2 ~ 36.0
2	Male	38	−8.75/−0.50x5 = 1.0	54 ~ 86	0.138	24.5 ~ 31.6
3	Female	31	−8.25/−1.75x175 = 1.0	58 ~ 217	0.396	22.7 ~ 27.4
4	Female	29	−3.75/−1.50x10 = 1.2	67 ~ 177	0.521	32.4 ~ 43.8
5	Female	38	−8.75/−1.00x165 = 1.0	54 ~ 134	0.998	35.5 ~ 85.8
6	Male	32	−1.50/−0.75x25 = 1.0	57 ~ 162	0.580	28.6 ~ 68.5
7	Female	38	−5.50/−0.50x145 = 1.2	76 ~ 144	0.588	20.4 ~ 23.2
8	Female	42	−5.00/−1.25x175 = 1.2	61 ~ 118	0.853	27.4 ~ 44.8
9	Male	26	−2.25/−0.75x65 = 1.2	50 ~ 189	0.441	38.9 ~ 70.9
9	Male	26	−2.25/−0.75x65 = 1.2	61 ~ 169	0.310	29.1 ~ 52.8

*CDVA* corrected distance visual acuity

**Fig. 2:**
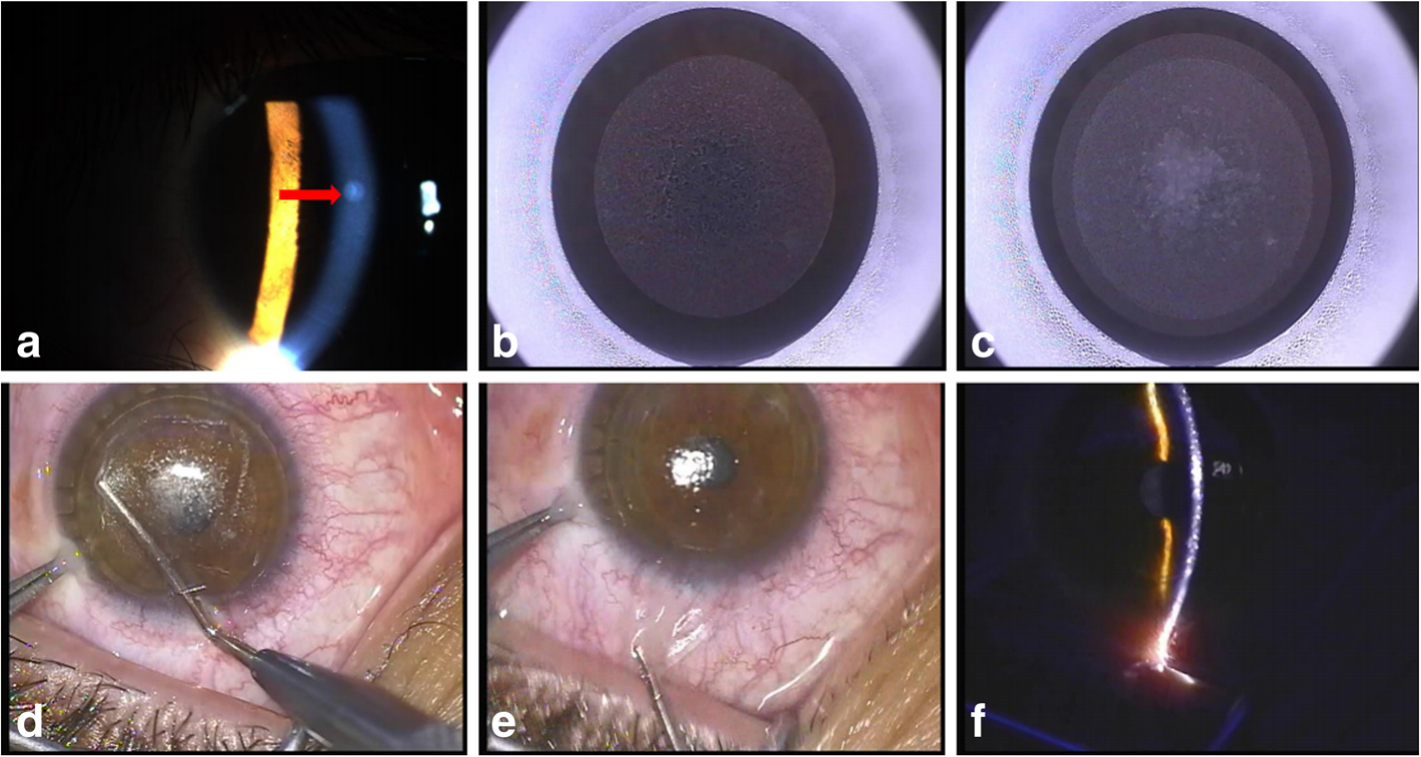
The preoperative, intraoperative, and postoperative corneal examinations in case No. 6. **a** The preoperative corneal opacity was noted by slit-lamp microscopy (*red arrow*). **b** The posterior surface of the lenticule scan. **c** The anterior surface of the lenticule scan. **d** The lenticule dissection. **e** The lenticule extraction. **f** The postoperative routine corneal examination using slit-lamp microscopy

**Fig. 3:**
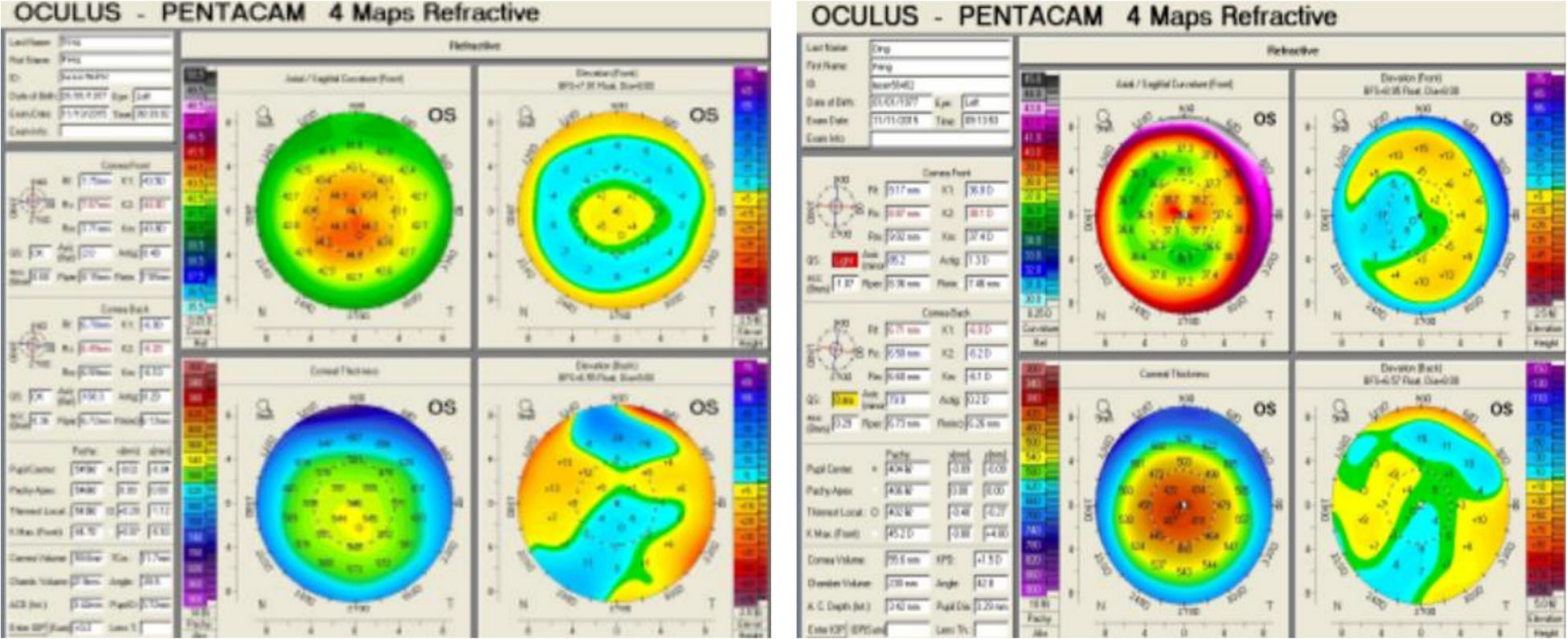
The corneal morphology before and after SMILE surgeries by Scheimpflug tomography system in case No. 6

**Fig. 4:**
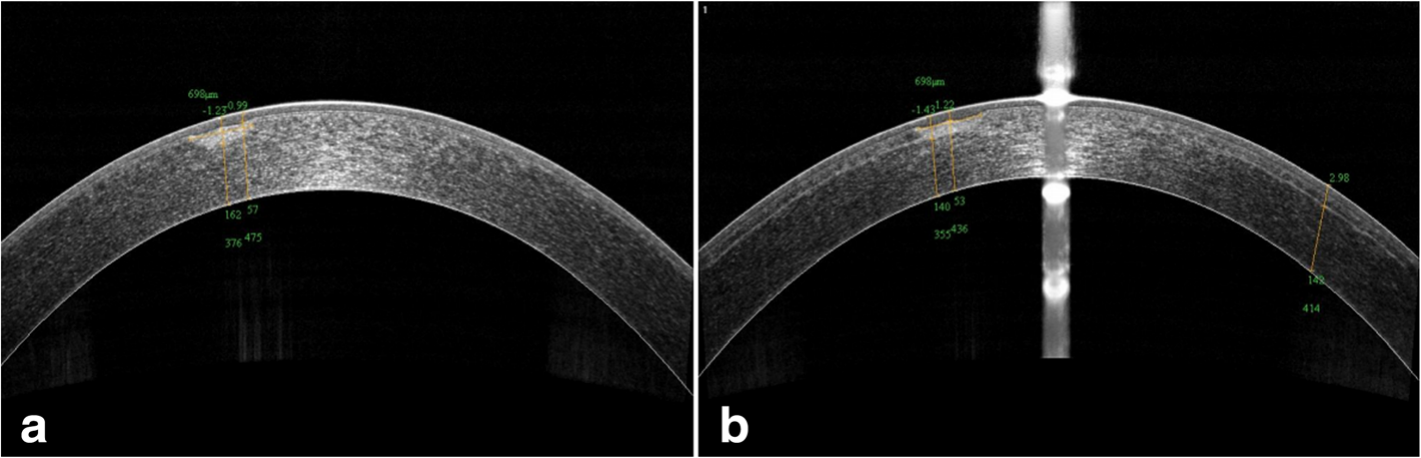
The preoperative and postoperative depths of corneal opacity were measured from AS-OCT images in case No.6. **a** It was 57 to 162 μm before surgery. **b** It was 53 to 140 μm after surgery

All surgeries were uneventful and there were no intraocular complications such as OBLs, black spots, suction loss, difficulties in dissection, or broken lenticules (Fig. 2). Moreover, there was no decentration following SMILE (Fig. 3). Figure 4 shows the morphology of corneal cap, which was good.

The safety (postoperative CDVA/preoperative CDVA) and efficacy (postoperative UDVA/preoperative CDVA) indexes were 1.10 ± 0.24 and 1.08 ± 0.16, respectively, at 1 month after surgery. The postoperative spherical equivalent (SE) was 0.27 ± 0.23 D; all eyes were within ±0.75 D and 8 eyes (88.9%) were within ±0.50 D.

Preoperatively, the corneal opacity in all patients was within the optical zone. The preoperative mean maximum density and depth in deepest location of corneal opacity were 48.5 ± 20.7 and 152 ± 38 μm, respectively. The mean maximum density of corneal opacity was 49.8 ± 26.7 at 1 month after surgery, and there was no significant difference in the value before and after surgery (*P* > .05). The mean depth in deepest location of corneal opacity was 117 ± 28 μm at 1 month postoperatively, and there was significant difference in the value before and after surgery (*P* < .05). The postoperative area of corneal opacity was smaller than the preoperative one (*P* < .05) (Table 2).

**Table 2:** The preoperative and postoperative mean density and depth in the deepest areas of corneal opacity and mean area of corneal opacity

Corneal opacity	Preoperative	1 day post-op	1 month post-op
Density	48.5 ± 20.7	50.6 ± 28.2^b^	49.8 ± 26.7^b^
Depth (μm)	152 ± 38	120 ± 25^a^	117 ± 28^a^
Area (mm^2^)	0.605 ± 0.330	0.507 ± 0.568^a^	0.511 ± 0.319^a^

^a^ *P* < .01

^b^ *P* > .05

## Discussion

The cornea was photodisrupted by FS with large number of small vacuoles to facilitate the procedure of cutting the corneal tissue [1]. The FS can penetrate the predetermined depth of clear cornea without a heating effect or impacting the surrounding tissue [12]. It is important to perform FS treatment with a transparent cornea, and is it possible to perform SMILE surgeries using VisuMax FS in the opaque cornea. The aim of our study was to demonstrate the effectiveness of these procedures.

Mian et al. [13] suggest that the ability of FS to photodisrupt corneal tissue is not hampered by optical haze, and Choi et al. [14] have reported a successful outcome after lamellar keratectomy using FS in a patient with a superficial corneal opacity. On the other hand, Tomita et al. [11] have shown that eyes in the IntraLase group had gas breakthroughs with corneal flaps of 100 to 130 μm thick. Seider et al. and Von et al. have demonstrated that there will be problems of lifting the flaps in patients with corneal opacities and scarring [15, 16]. Corneal opacities or scars can affect femtosecond laser operation because of a defect in Bowman’s membrane in patients with corneal opacities [17, 18]. Unlike FS in laser-assisted in situ keratomileusis(LASIK), the corneal tissues must be photodisrupted by FS laser twice in the SMILE procedure. So far, there is no related research regarding quantitative measurement of the density of corneal opacity in FS treatment. Mohammad et al. [19] have quantitatively measured the density of corneal opacities in patients treated with phototherapeutic keratectomy (PTK), and the results showed a good correlation between the degree of the corneal opacity and its density as measured by the Scheimpflug camera system.

In the present study the opacity of corneas were within optical zone in all patients. Based on preoperative OCT and Pentacam results, the patients gave their informed consent and agreed that if the laser scan was not completed during the SMILE procedure, alternative refractive surgery would be chosen. Finally, all patients successfully completed the SMILE surgery, with no black spots, OBLs, or other complications. It may be added that corneal opacities with an optical density value range of 20.4 to 85.8 could be penetrated by the VisuMax FS system in the course of the SMILE procedure. The 180 nJ of energy and 4.5 μm of spot-and-track spacing distance was used for lenticule and cap scanning, representing a higher-energy and greater spacing distance. It may therefore be helpful in penetrating opaque corneas and diffusing the microbubbles produced during FS scanning. Therefore we don’t think it is necessary to change the cap thickness. All surgeries were uneventful without any intraoperative complications including epithelial abrasions, small tears at the incision site, OBLs, or difficult lenticule extractions [20].

Our outcomes show that the SMILE procedure is the safe and effective in treating patients with corneal opacities of a determinate density that are not within the central cornea. In our study no postoperative complications such as trace haze, interface inflammation secondary to central abrasion, or diffuse lamellar keratitis were observed [21]. The postoperative densities of the corneal opacities were reduced after SMILE because the extracted lenticules included opaque cornea. However, there was no significant difference in terms of density of corneal opacity before and after the SMILE procedures, showing that the corneal opacity remained after surgery. Moreover, we measured the depths of the corneal opacities using AS-OCT and the results were repetitive. Consequently, it is feasible to screen patients with corneal opacities for SMILE surgery using the Scheimpflug camera system and AS-OCT.

Further research is needed to determine whether a corneal opacity affects long-term visual quality. The correlation between surgical parameters and the density of a corneal opacity should be demonstrated in further studies, which need to be conduct with large number of patients.

## Conclusion

In conclusion, our study shows that patients with corneal opacities can successfully be treated with the SMILE operation. The short-term outcome is good, however the long-term outcome requires further study.

## Abbreviations

AS-OCT: Anterior segment optical coherence tomography
CDVA: Corrected distance visual acuity
D: Diopter
FS: Femtosecond laser
IOP: Intraocular pressure
LASIK: Laser-assisted in situ keratomileusis
OBL: Opaque bubble layer
PTK: Phototherapeutic keratectomy
SE: Spherical equivalence
SMILE: Small incision lenticule extraction
SPSS: Statistical package for social sciences
UDVA: Uncorrected distance visual acuity
W-W: White-to-white
